# Supplementary material for: Provider perspectives on empirical antibiotic treatment for tuberculosis-like symptoms in South Africa’s private general practice sector: A qualitative study in two cities
Source: PLOS Glob Public Health. 2025 Jun 24;5(6):e0004742. doi: 10.1371/journal.pgph.0004742 (PMC12186942; doi:10.1371/journal.pgph.0004742)
Supplement: S1 Text — (DOCX) [file pgph.0004742.s001.docx]

**Semi-structured provider interview guide**

1. **Introduction and consent taking**
   1. Take a moment to remind them about the earlier study and that we want to learn a bit more about their experience to be able to contextualise the findings based on the simulated patient interactions.
   2. Try to get a sense of how much time they have for the interview.
   3. Explain that you are a social scientist and may not always be familiar with technical or clinical terms. Ask that they bear with you if you ask for clarification on some terminology.
2. **Let us talk a little about your practice**.
   1. Please describe to me the typical demographics of your patients.
   2. In an average week, what proportion of your patients are new (first-time)?
   3. What do you think brings patients back to your practice?
   4. What are some of the challenges you face with regard to having patients return to you?
   5. What makes you particularly proud of your practice?
   6. Please share with us/me what you enjoy about having a private practice in this community?
   7. Conversely what has frustrated you about having a private practice in this community.
   - If you could strengthen practice for GPs in <Durban/this community>, where would you start?
3. **We would like to learn a bit more about the typical complaints you see in your practice.**
   1. What are some of the most common conditions you encounter?

(probe any conditions that may overlap with TB symptoms, e.g., flu, bronchitis, asking how doctor typically approaches treatment, what about patients who present with more general symptoms)

What are some of the conditions you see that are more challenging to diagnose? Why is this the case?

- 1. What conditions do you think are best serviced by the private sector? Why?
  2. Conversely, what conditions do you think are best treated in the public sector? Why?

1. **We’d now like to chat a bit about tests that you offer.**
   1. What are the common tests you usually offer patients on site? (does this differ between cash paying *vs medical aid patients)*
      1. If you send patients off site where do you usually send them to?
      2. Are there specific tests that you would refer them to a public facility for? (Does this differ between cash paying and medical aid patients?)
   2. What about HIV testing
      1. How do you approach this subject with a patient?
      2. Is there a different approach you have with a new patient vs a regular patient?
      3. Do any other factors guide your approach such as the patient’s 5?
      4. How often do you see HIV in practice? (What proportion of your patients are positive)
2. **How about medications. Do you dispense medications yourself?**
   1. What are the common reasons for you to prescribe antibiotics – what are your favourite ones for respiratory conditions?
   2. What are the common reasons you would order a steroid?
   3. What about injections – what injections do you usually offer patients on site?
   4. We have heard that patients expect to leave a doctor’s visit with medicines in hand – what is your opinion about this? How do you balance this with giving out potentially unnecessary medicines?
   5. On a typical day, what proportion of your patients would you prescribe medications to (probe: mostly all, seldom)?
3. **Let us talk about making decisions and acting with respect to presumptive TB.**
   1. How often do you see TB in your practice? (in the last 6 months how many people with TB did you see)
   2. What are some of the factors you consider before you suspect TB? (probe for auscultation)
   3. What are the challenges to diagnosing TB (*probe for generalised symptoms, more common diagnoses for these symptoms, complications involving HIV*)?
   4. What are the challenges to treating TB (probe referral to public sector, cash paying vs medical aid patients, and patient preferences)?
   5. There is a concept you may be familiar with called the TB cascade of care. The concept describes how a patient with TB may get lost in the healthcare system at various points before they are diagnosed or treated adequately. Part of the concern is the number of times a patient must make contact with the health system, especially given that people with TB are more often of low socio-economic status. As a provider, how might you address this concern?

**7 . Some of the work we do is around TB and HIV management. So we would like to round off by asking about your involvement in continuing medical education (CME), generally, or your or needs in this respect.**

1. What are your CME needs specifically for TB-related diagnosis and management?/Are there particular aspects of TB that you would like to know more about? How about drug-resistant TB?
2. What are your CME needs regarding HIV diagnosis?
3. **Is there anything else you would like to add?**

Thank you very much for your time.
