# Supplementary material for: Provider perspectives on empirical antibiotic treatment for tuberculosis-like symptoms in South Africa’s private general practice sector: A qualitative study in two cities
Source: PLOS Glob Public Health. 2025 Jun 24;5(6):e0004742. doi: 10.1371/journal.pgph.0004742 (PMC12186942; doi:10.1371/journal.pgph.0004742)
Supplement: Attachment — Submitted filename: PGPH-D-24-02250-1_16Nov24.pdf [file pgph.0004742.s003.pdf]

PLOS Global Public Health

Provider perspectives on empirical antibiotic treatment for tuberculosis-like symptoms in South Africa's private general practice sector: A qualitative study in two cities

--Manuscript Draft--

**Manuscript Number:**	PGPH-D-24-02250
**Article Type:**	Research Article
**Full Title:**	Provider perspectives on empirical antibiotic treatment for tuberculosis-like symptoms in South Africa's private general practice sector: A qualitative study in two cities
**Short Title:**	Private general practitioners' perspectives on empiric antibiotic treatment of TB symptoms
**Corresponding Author:**	Jeremiah Chikovore, PhD<br>Human Sciences Research Council<br>SOUTH AFRICA
**Order of Authors:**	Jeremiah Chikovore, PhD
	Jody Boffa, PhD
	Sizulu Moyo, MBChB, PhD
	Angela Mak
	Zimasa Gavu, MPH
	Angela Salomon, MD, PhD
	Madhukar Pai, MD, PhD
	Amrita Daftary, PhD
**Keywords:**	quality of care; Tuberculosis; low- and middle-income countries; private practice; Qualitative; South Africa
**Abstract:**	South Africa has among the highest tuberculosis (TB) burden globally. While TB is commonly treated in the public sector, some first seek care in the private sector. Recent research on quality of TB care has shown that in South Africa, private general practitioners (GPs) perform less well than do public sector care providers in terms of TB testing and drug-dispensing practices. We aimed to describe GPs' decision-making practices related to empiric antibiotic treatment when presented with TB-like symptoms to inform potential avenues for intervention. Within a larger study on private sector TB management in two cities, eThekwini and Cape Town, we qualitatively interviewed a purposive sample of 30 GPs, 15 per site. The participants varied in how well TB and HIV were managed in the parent study, gender, age, and practice community. The data were analysed through coding and constant comparison. The GPs acknowledged that treating respiratory symptoms with broad-spectrum antibiotics was a common practice. While some identified the use of antibiotics to treat presumed bacterial infections on the basis of experiential insight, others deemed it a best practice to rule out other infections prior to a perceived inconvenient private laboratory or public sector TB test. Additionally, management decisions seemed susceptible to perceived or expressed pressure from clients who may expect on-the-spot treatment. Some GPs indicated using the practice to reduce the financial burden on patients perceived to be of lower socioeconomic status. Empirical antibiotic treatment for TB-like presentations in South Africa's private sector could be explained by the absence of accessible and affordable TB and general bacteriologic tests at the point of care, leading GPS to, among other, seek to 'rule out' possible bacterial infection. Potential interventions must consider the complex context for GPs and, simultaneously, increasing the salience of inappropriate antibiotic use, heightening GPs' suspicion index for TB, and implementing public-private partnerships that can make rapid molecular testing services available to clients of private sector GPs.
**Additional Information:**	
**Question**	**Response**

Powered by Editorial Manager® and ProduXion Manager® from Aries Systems Corporation

**Financial Disclosure**<br><br>Enter a financial disclosure statement that describes the sources of funding for the work included in this submission and the role the funder(s) played. This includes grants and any commercial funding of the work or authors.<br><br>This statement will be typeset if the manuscript is accepted for publication.<br><br>*Please review the submission guidelines and the instructions link below for detailed requirements and guidance.*	This work was supported in part by the Bill & Melinda Gates Foundation [Grant Number OPP1172634]. Under the grant conditions of the Foundation, a Creative Commons Attribution 4.0 Generic License has already been assigned to the Author Accepted Manuscript version that might arise from this submission. The funder had no role in the study design, data collection, data analysis, data interpretation, or writing of the report.
**Competing Interests**<br><br>On behalf of all authors, disclose any competing interests that could be perceived to bias this work.<br><br>This statement will be typeset if the manuscript is accepted for publication.<br><br>*Please review the instructions link below and PLOS Global Public Health's competing interests policy to determine what information must be disclosed at submission.*	JC, JB, SM, and AS have published with Madhukar Pai and Amrita Daftary in the past 5 years.
**Data Availability**<br><br>Before publication, Authors are required to make fully available and without restriction all data underlying their findings. Please see our PLOS Data Policy page for detailed information on this policy.<br><br>A **Data Availability Statement**, detailing where the data can be accessed, is required at first submission. Insert your	All data files used for the manuscript are available and will be accessible through The Human Sciences Research Council's data repository upon acceptance of this submission.

Data Availability Statement in the box below.<br><br>Please see the data reporting section of our submission guidelines for instructions on what you need to include in your Data Availability Statement.<br><br>This statement will be typeset if the manuscript is accepted for publication.<br><br>*PLOS allows rare exemptions to address legal and ethical concerns. If you have legal or ethical restrictions, please use the box below to detail these in full sentences for the Journal team to consider.*	

Powered by Editorial Manager® and ProduXion Manager® from Aries Systems Corporation

Manuscript

Click here to access/download;Manuscript;Manuscript_Chikovore_01Oct202

Provider perspectives on empirical antibiotic treatment for tuberculosis-like symptoms in South Africa's private general practice sector: A qualitative study in two cities

Short title: Private general practitioner perspectives on empiric antibiotic treatment of TB symptoms

Jeremiah Chikovore[1], Jody M Boffa[2], Sizulu Moyo[3,4], Angela Mak[5,6,] Zimasa Gavu[1], Angela Salomon[7], Madhukar Pai[7,8], Amrita Daftary[6,9]*

Authors affiliations:

1. Human Sciences Research Council (HSRC), Public Health, Society and Belonging (PHSB) Division, Durban, South Africa,
2. The Aurum Institute, Johannesburg, South Africa
3. HSRC, PHSB Division, Cape Town, South Africa
4. University of Cape Town, Cape Town, South Africa
5. Cumming School of Medicine, University of Calgary, Calgary, Canada
6. Dahdaleh Institute of Global Health Research, York University, Toronto, Canada
7. McGill University Health Centre, McGill University, Montréal, Canada
8. Department of Global and Public Health and McGill International TB Centre, McGill University, Montréal, Canada
9. School of Global Health, York University, Toronto, Canada
10. Centre for the AIDS Programme of Research in South Africa (CAPRISA), University of KwaZulu-Natal, Durban, South Africa.

Corresponding author:

* Email: adaftary@yorku.ca **(AD)**

Word Count:

Abstract: 318

Main text: 6374

References: 64

Abstract

South Africa has among the highest tuberculosis (TB) burden globally. While TB is commonly treated in the public sector, some first seek care in the private sector. Recent research on quality of TB care has shown that in South Africa, private general practitioners (GPs) perform less well than do public sector care providers in terms of TB testing and drug-dispensing practices. We aimed to describe GPs' decision-making practices related to empiric antibiotic treatment when presented with TB-like symptoms to inform potential avenues for intervention. Within a larger study on private sector TB management in two cities, eThekwini and Cape Town, we qualitatively interviewed a purposive sample of 30 GPs, 15 per site. The participants varied in how well TB and HIV were managed in the parent study, gender, age, and practice community. The data were analysed through coding and constant comparison. The GPs acknowledged that treating respiratory symptoms with broad-spectrum antibiotics was a common practice. While some identified the use of antibiotics to treat presumed bacterial infections on the basis of experiential insight, others deemed it a best practice to rule out other infections prior to a perceived inconvenient private laboratory or public sector TB test. Additionally, management decisions seemed susceptible to perceived or expressed pressure from clients who may expect on-the-spot treatment. Some GPs indicated using the practice to reduce the financial burden on patients perceived to be of lower socioeconomic status. Empirical antibiotic treatment for TB-like presentations in South Africa's private sector could be explained by the absence of accessible and affordable TB and general bacteriologic tests at the point of care, leading GPS to, among other, seek to 'rule out' possible bacterial infection. Potential interventions must consider the complex context for GPs and, simultaneously, increasing the salience of inappropriate antibiotic use, heightening GPs'

suspicion index for TB, and implementing public-private partnerships that can make rapid molecular testing services available to clients of private sector GPs.

Keywords*:* Quality of care; Tuberculosis; Low- and middle-income countries (LMICs); Private practice; Qualitative; South Africa

Introduction

[illegible]th Africa is among the 30 countries with the highest burdens of multidrug-resistant (MDR) TB and, given the high prevalence of HIV (16.3% among adults ≥15 years) (1), HIV-associated TB (2). In 2022, TB incidence overall, and incidence for drug-resistant TB, and among people living with HIV (PLHIV) was estimated at 280 000, 11 000, and 152 000, respectively (3).

Managing TB requires early identification and effective treatment to prevent health sequelae, transmission, distress, and economic hardship (4). The World Health Organization (WHO) recommends that people with pulmonary TB symptoms be tested using nucleic acid amplification tests, e.g., Xpert Ultra (5), and receive standardised anti-TB treatment (ATT) if they test positive (6, 7). Those who test negative but have significant exposure to a person with TB should receive TB preventive therapy. In South Africa, Xpert Ultra is used as the standard first-line test [illegible]However, a TB diagnosis can be made and ATT initiated in the absence of microbiological confirmation, based on symptoms, chest X-ray results, comorbidities especially HIV, and/or a lack of response to broad-spectrum antibic[illegible]Among PLHIV, guidance urges routine TB testing, ATT for those found to have active disease, and preventive treatment for those without active TB (9, 10).

Detection of people with TB in most high TB burden countries, including South Africa, has typically relied on passive case finding (11, 12), whereby people present to the health sector when symptomatic. The inadequacies of this approach are well described (13). Some people with TB-like symptoms seek care from private healthcare providers, who may offer levels of care that vary from National TB Programme (NTP) or international guidelines and standards (4). A patient pathway analysis of five high TB burden countries revealed that up to

60% of people with TB began by seeking care in the private sector (14). In South Africa, nearly one-third of households first seek care from private doctors and hospitals (15). Although many people may thereafter move into the public sector, the private sector continues to provide primary care services to 28–38% of people (16).

Thus far, however, limited attention has been given to the quality of care in South Africa's private health sector (17, 18). There is little understanding of private provider decision-making processes and of the challenges faced and strategies used to overcome these challenges (17, 18). This may be a result of expectations or assumptions that the extens[illegible] resources in the sector (19, 20) ought to translate into person-centred and high-quality care. We sought to fill this knowledge gap by exploring and describing the dynamics of care provision for people presenting with TB-like symptoms (prolonged cough, fever, drenching night sweats, and/or unintended weight loss) among private general practitioners (GPs) in South Africa, with a special focus on empirical antibiotic use.

In a previous study, we used the standardised patient methodology to describe the quality of TB and HIV-associated TB diagnostic care and quantify the know-do gap (i.e., difference between intended and observed practices) among 212 private GPs practising in high TB burden urban areas of eThekwini and Cape Town, South Africa (21, 22). Analysis of the quantitative data emanating from 511 unannounced visits from trained standardised patients (akin to mystery shoppers) revealed several missed opportunities to appropriately investigate or refer for both TB and HIV testing (21). Despite reporting symptoms suggestive of TB, including in some cases, producing a confirmed laboratory report, appropriate testing, referral, or treatment were not initiated in more than 50% of interactions. Limited efforts were made to inquire about HIV status, despite policy support for TB-HIV service integration

given the dual disease burden (23, 24). Notably, gaps were found between what providers thought they should do (as tested through vignettes) and what they actually did (as tested through their management of standardised patients), measuring 37% for TB patients and 18% for HIV patients (21). These know-do gaps suggest that factors other than knowledge likely affect care practices. The analysis also revealed that medicines were dispensed or prescribed in almost 90% of interactions, at 3.1 (SD=1.6, range 0–8) medications per visit, with antibiotics being dispensed or prescribed in 77% of inte[illegible]ons. Other common medications which included cough syrups (20.7% of all medicines) and analgesics (12.9% of all medicines) are primarily to abate symptoms (21, 22). The present qualitative sub study sought to enhance insights into the contextual dynamics of antibiotic use by private GPs, specifically, in the care provision for people with TB-like symptoms.

Materials and methods

Design, selection of participants, and data collection approach

The parent study took place in eThekwini and Cape Towns from August 2018–July 2019 and is described in detail elsewhere (21, 22). Subsequent to unannounced visits from standardised patients in each city, a subsample of consenting GPs was recruited and privately interviewed using qualitative methods to elicit deeper meanings and understanding of the contexts of consultation considerations. GPs were purposively chosen to include both those who provided correct management of TB and/or HIV care according to national guidelines and as defined in the parent study (i.e., referred for a TB test or to the public sector for further care and/or inquired about HIV status or initiated an HIV test) and those who did not, during standardised patient interactions. The participants were further chosen to achieve variation

by age, sex, and location (areas likely to have patient profiles from different races and socioeconomic groups) to obtain the breadth of dimensions to the topic (25). A sample of 30 GP participants was sought due to feasibility and an expectation to achieve saturation of relevant themes (26).

The interviews took place at GPs' practices and lasted approximately 30 minutes. The questions were based on an open-ended guide generated from the study goals and leads from a preliminary analysis of the larger study. Subsequently, typical of qualitative research approaches, questions were continually reviewed to allow the pursuit of crucial emerging leads (27, 28). (The core guide is included as a supplemental file). In addition to background information about the practice (such as client demographics, challenges commonly encountered as well as positive aspects about the practice, and typical complaints seen), the guide covered the following interrelated topics: i) mechanisms of decision-making around empirical treatment in response to respiratory symptoms; ii) accompanying actions and underlying rationale for presuming or not presuming TB; and iii) consideration, if any, given during consultation to a patient's contextual (social, economic, familial) circumstances. Research assistants (n=5) from each city were trained to conduct qualitative interviews, including role-play and nonevaluative approaches to probing. The participants were not informed of their actual practices observed during standardized patient interactions in the main study, although the interview responses were also analysed in the context of those observations (see below).

Ethics approval and consent to participate

Ethics approval was granted by the Human Sciences Research Council Ethics Committee (HSRC Ref#: 2/18/10/17) and the McGill University Ethics Board (MUHC Ref#: 2018--4390). Informed consent was sought and obtained from all participants, and anonymity and confidentiality were maintained throughout the research process. The interviews were audio-recorded with the participants' consent.

Data management and analysis

Preliminary analysis, involving debriefing between lead researchers and research assistants, closely tracked data collection in each city, starting with eThekwini, and enabled immediate data review (28, 29) and adjustment of questions to pursue emerging leads or sharpen focus. The recordings were transcribed and translated verbatim, checked for accuracy, and analysed both inductively and deductively (30, 31). The data were initially coded (32) in large chunks on the basis of the study objectives and preliminary analysis of the larger study. Through emergent identification of their properties, or alternatively, through the use of existing guide sub-questions, the large chunks were further broken down to identify finer dimensions, in a process that continued until no further breakdown could be performed. Relationships between codes and concepts were identified and refined through constant comparison (33). Text was then retrieved by their codes/subcodes, read critically, and further interpreted in the context of participants' mea[illegible]d practices, the study settings, and the wider TB epidemiological and policy milieu (34, 35). At this stage, the data were recoded and reconnected in more abstract ways to generate high-level themes, which we present below. The handling and retrieval of data and coded chunks of text were facilitated with the use of

NVivo qualitative analysis software version 12 (QSR International). JC led the coding and analysis, with JB and AD providing confirmatory/alternate perspectives. The analysis was further shared, debated and refined over repeated consultations with the full team. To maintain participant confidentiality, representative quotes presented ahead are labelled only with gender, approximate age, and measured practice. The study is presented according to COREQ (consolidated criteria for reporting qualitative research) guidance for reporting qualitative research (36).

Findings

Overview of GPs and their practices

The GPs in the main study were drawn from selected high-TB burden communities in two cities (21). All GPs approached for interv[illegible] consented: 15 were from eThekwini (2 F, 13 M), an[illegible] were from Cape Town (3 F, 12 M). The providers' characteristics are described in Table 1. Their sociodemographic characteristics were similar to those of the main study (21). Their practices were also aptly heterogeneous to enable qualitative anal[illegible] The conditions that different GPs reportedly commonly saw and managed, ranged from the common 'flu' to colds, fever, and headaches, and, depending on a facility's size, chronic issues. The GPs described thriving on and building their reputations through managing a range of health issues including geriatric conditions, arthritis, hypertension, and diabetes, and referring complicated ones. The patients seen were generally described as of varying ages and from diverse geog[illegible]cal locations and communities. GPs also varied in the range of their onsite services, with some offering chest X-ray and sputum sample collection services, and medicines ranging from analgesics to antipyretics and a variety of antibiotics, and others

having no onsite testing or treatment supplies. Others had ‘IV’ (intravenous) and ‘casualty’ (emergency room) equipment onsite. GPs who kept and dispensed drugs on site seemed more prone to using empirical treatment.

Table 1: **Provider characteristics**

Characteristic	Overall n=30	eThekwini n=15	Cape Town n=15
Sex, n (%)			
Female	5 (16.7)	2 (13.3)	3 (20.0)
Male	25 (83.3)	13 (86.7)	12 (80.0)
Area, n (%)			
City	8 (26.7)	8 (53.3)	0 (0.0)
Township	8 (26.7)	6 (40.0)	2 (13.3)
Suburb	14 (46.7)	1 (6.7)	13 (86.7)
Years in practice, median (IQR)	24 (13-33)	23 (13-33)	25 (12-33)
Daily patient load, median (IQR)	25 (16-30)	27.5 (15-45)	25 (16-30)
Consult fee in ZAR, median (IQR)	350 (280-355)	330 (280-250)	350 (260-380)

In the rest of the findings, we present three interrelated themes related to general care provision by GPs. The first addresses the dynamics of antibiotic prescription, focusing on infections they deemed worthy of treatment with antibiotics, as well as their use of empirical treatment and determination of differential diagnoses. The second theme is about the susceptibility of GPs’ management decisions to pressure they perceived or that was expressly

signalled from patients. The final theme probes the role that economic and financial considerations play in the context of GP practices. (Figure 1)

[Insert Figure 1 here]

Antibiotic prescription dynamics

GPs mentioned that conditions they held to be complex and/or chronic conditions such as diabetes, HIV, hypertension, ischaemic heart disease, TB, cholesterol, trauma or ectopic pregnancy require onwards referral. Such conditions were described as entailing expensive management regimens, with some being easier or better managed by the public health system. The size of their facility, onsite availability of suitable equipment, and mindfulness about private management costs for clients were among the GPs' considerations when referring out the conditions they deemed chronic or complex. Conversely, conditions viewed as 'minor' such as respiratory conditions indicative of bacterial infection, or relating to 'flus' and colds, could be treated with a broad-spectrum antibiotic at the point of presentation. (In some settings, including South Africa, common parlance addresses 'flu' interchangeably with influenza-like or cold-like symptoms, and there is often no distinction between 'flu' and 'cold' (37). This reflected in how the GP participants frequently mentioned 'common flu and colds' in one breath. The GPs described assessing presenting signs and symptoms to determine a course of action and the timing of treatment initiation. Determining symptom severity, making differential diagnoses, and choosing what action to take emerged as being situationally grounded, pragmatic, and heavily reliant on a specific GP's balancing of

their experiential insight, immediate reading of presenting symptoms, and consideration of patient history.

> *"With infections, as an experienced doctor, you know this injection doesn't work; this one works. … If a patient has a flu, you don't give them [an antibiotic]… body pain you might give [an] anti-inflammatory. However, if they've got a bad infection in the chest, you give them an antibiotic ….”* (Male, aged in the middle-50s)

> *"If I suspect viral [infection] … I'll probably give something just to clear the symptoms…the cough …the nasal congestion … kill the sore throat … but if I think there is secondary bacterial infection -- (for viruses, antibiotics don't work) -- I give antibiotics (**which ones?**) Penicillin … works all the time … some people react, but I will inquire about that first."* (Male, aged 30 years)

Without laboratory test results, GPs recognised that their medical decisions were innately infused with uncertainty. In light of this, they report giving antibiotics to avoid burdening clients with multiple trips or in an attempt to eliminate the potential risk of transmission to social networks (particularly where their clients generally lived in crowded conditions), or to prevent the worsening of outcomes from conditions that, while not yet confirmed, might just fully or partially resolve with antibiotic treatment.

> *"Again… we don't have resources to test for [bacterial] infection [on site] … but I bet you this patient is going home … fifteen people in one house… chances of them getting contact with any bacteria (the patient might have) are very high… are you going to say, 'Let me take your sputum now and test if you truly have an infection, and you come back (for results)?' … it's not going to work …*" (Male, aged 55 years)

"Instead of giving them a script to a pharmacy … most cash [paying patients] wouldn't be happy about it … I mean the sooner you think about the GP practices: you cannot confirm with each patient exactly what sort of bacteria… we don't do microbiology … you can't do sputum and swabs on everyone; you sort of use your discretion … and cover in broad (terms) what you (think you) can." (Male, aged 50s)

Empiric treatment was commonly viewed and employed as a stopgap measure that aided diagnostic and decision-making steps. The GPs' accounts indicated that such empirical treatment, including antibiotics, was widespread. Treating and observing was viewed as part of a systematic-looking approach, typically beginning with broad and/or low-strength/lower-dose antibiotics, then increasing potency or narrowing microbial targeting as perceived necessary, and ultimately ordering investigative tests or referring if needed. During this process, progress was reviewed over time periods ranging anywhere between three days and four weeks.

"Mostly you work systematically… best is to treat the basic things ... cover broadly … use an antibiotic injection and give them some oral antibiotic… So, say I was treating for … bronchitis, if you are not improving after 5 days, I've given you a strong antibiotic, I've given you an injection, the next thing let's do a sputum, let us do an X-ray... always do the basic things, then advance or refer after." (Female, aged 30s)

Sometimes, however, GPs empirically treated with strong antibiotics '*because when people (eventually) come… they have exhausted the [over-the-counter] things they get from the pharmacy … [and] that obviously hasn't worked…"* (Female, aged 50s).

Since our work was nested within a TB-focused study, we probed GPs' considerations about TB. GPs highlighted the need for prudence and for treating and observing rather than

alarming clients with a differential diagnosis that was, in effect, confirmable only by the additional step of testing. Building on the notion that empirical treatment was 'systematic', GPs repeatedly impressed that they were guided by thorough examinations and history-taking when deciding on management options. Some described deferring consideration of TB for weeks until symptoms persisted or empirical treatment had failed to elicit a positive response. Yet others described the role that relatives sometimes play in contributing to history-taking through clarifying symptoms and illness history.

> *"(If) their mark is suggesting bacterial... obviously they need an antibiotic, so we give them… usually something penicillin-based …and obviously if it's not improving -- you know, once we hit the two-week sort of barrier -- then you worry about TB."* (Female, aged mid-30s)

> *"Most come saying, 'I'm losing weight'. They won't say 'I'm coughing and have been sick for a while' … Until the wife, son, or mother declares 'No… it's been like this for 3 months now.' Right, then we examine them, and say, 'Listen, this is most likely TB. It could also be a normal chest infection. We give you a 3-day course and see what happens in 3 days.'"* (Male, aged 60s)

In principle, GPs seemed aware of recommended best practices but revealed notable contradictions when describing general practice, and a weaving between what may be described as 'appropriate' and 'inappropriate' management actions. One GP (male, age 50s) related, for instance, that he 'tested' prior to giving any medicines but proceeded to say, if not yet suspecting TB, he might give '*treatment for bronchitis, and if … not getting better … send to another differentiating diagnosis used to exclude TB*'. He later said he considered any prior treatment received by the client elsewhere, alongside presenting symptoms. He then reiterated a commonly mentioned reliance on observation and examination to make a first

call, saying '*just by looking at a patient… (one) can tell something is not right',* despite stating, in the same interview, that a person can have TB and look 'normal'. Assumptions about the socioeconomic standing of presenting clients, as described below, also affected GPs' management decisions.

More GPs described treating before investigating -- regardless of whether they considered TB on the basis of symptoms -- and waiting a few days ('72 hours') to review for '*more definitive symptoms.*' An effusion or severe deterioration would then prompt immediate referral for chest X-ray and other tests (illustratively, two male GPs, one aged 60 years and another aged in the 40s, expressed these sentiments). Treating and reviewing was said to be necessary '*before worrying about TB*' because TB was considered to be '*the big one*' (female, aged in the middle of their 30s). GPs also mentioned treating other urgent symptoms first (e.g., 'tight chest' or bronchitis), even in the presence of typical TB-related symptoms, on the understanding that, by this stage, the client had already developed pneumonia.

Notably, as much as GPs might consider TB at the outset, they nevertheless drew on a wide interpretation of the national and international guidelines for antibiotic use in making decisions around and investigating for TB.

> *"With TB, you'll suspect from the [outset]. They'll come to you and say 'I've used everything… but now it's been 3 weeks or … a month. … I'm losing weight, … appetite... feeling weak. Now I am starting to cough blood, I'm having night sweats,' and you're thinking,* ***Okay I think I should proceed (as TB)*** *… But the protocol is at least I should give antibiotics for a week, any simple infection from pneumonia, bronchitis and everything should clear on good antibiotics. 'After a week come back so that we assess you again.' (if) still unwell, from there, I must do a (referral) letter."* (Male, aged 30s)

Balancing perceived expectations of clients

Clients were frequently portrayed as exerting pressure on GPs to be managed in certain ways. A common sentiment was that, because they paid for the consult, they had certain expectations from the consultation and this included that they would receive medicines.. Providers also felt that they needed to make an effort to accommodate these expectations, especially for clients paying cash, and also, to the extent possible, minimize difficulties and inconveniences to patients while seeking healthcare. The GPs also indicated that some clients remained reluctant to undergo TB investigations, even after being treated with no improvement over a lengthy period. One GP described trying to convince clients that they *'wait first, 3, 4 weeks for improvement (after first treating with antibiotics) … if none and you're on medical aid (health insurance), go for TB X-rays. [But] some you truly must convince them that they must do this'* (Male, aged 45--55 years), while others recounted how some clients would declare themselves as having negligible risk for TB ('*I am wealthy, I don't get TB*').

The GPs suggested that they resisted the pressures in several ways. One was to clarify upfront their role 'to treat the patient to the best of [their] knowledge and based on the patient's real needs' (male, aged in their late 50s). They could also give the impression they were yielding and cooperating by prescribing medications that were relatively benign and '*not harmful.'* Another way was to restress nonpharmaceutical measures, such as advising clients that talking to the doctor was in itself a form of treatment or intervention, or, for illnesses such as flu, recommending rest, vitamin supplementation, nasal decongesting, and analgesics to manage headaches and fever. GPs could also more explicitly remind users of risks associated

with unnecessary use of medicines; they recounted harnessing an authoritative stance, which was also expected of them, to promote these recommendations.

> *"…speak to them strongly that we don't want to cause resistance because that's a big thing in our country…. They may not like it, but you've got to be firm … 'next time you're sick the injection won't work if you take it for every flu and small thing.'" (*Female, aged 30s)

In isolated instances, GPs indicated that they indeed yielded, noting the need to meet clients' expectations or to help 'boost' the body's capacity to fight disease.

> *"A lot of my patients expect injections … antibiotics … so they get [them]. … It is patients' expectations… we give in to it."* (Male, aged 50s)

> *"Most people love injections, especially the elderly. [I commonly give] flu medications… pain injection ... just boost them up … umm antibiotics like the broad-spectrum ones have severe side effects, like they need an IV … I'll just give them the pure based; I will give them penicillin … [I give antibiotics for] ear, throat and nose infections, the chest infections; umm bowel infections not as much… umm urinary tract infections, skin infection … ones I know will not resolve by themselves …. the body needs a bit of help. [It] must do the most part, but antibiotics help a little bit."* (Male, aged 30s)

Economic and financial considerations in general practice decisions

Accounts indicated that finance-related matters influence the course of patient management. GPs saw diverse clients, many of whom were economically disadvantaged, and they described feeling obliged to serve them compassionately. This meant that socioeconomic factors were a key consideration for where to refer for tests or further management, or which drugs to prescribe. Moreover, as indicated earlier, chronic or complex conditions were regarded as expensive to manage, particularly in the private sector; GPs

described taking this into account when making referral decisions for uninsured clients. Some even mentioned that they did not turn away clients who were short of money lest a serious event ensued; rather, they treated them ‘at cost’ (supposedly, without making the slightest profit), or they even elected to carry the losses associated with dispensing medicines below what these had cost them. GPs viewed their decision to stock and dispense medicines on site to minimise patient inconvenience.

Several GPs advised clients to seek (further) attention at public facilities, recognising that this would be their most financially viable option since public care is free for those earning below a certain level or without income. However, balancing their goodwill against financial implications for their practice was not without tension.

> *“In general, we try to give our patients the facility of getting everything here and save them the trouble of getting medicines from the chemist… There are some who do not always have transport … we tend to be compassionate towards the patient.”* (Male, aged 60s)

> *“Look, the medication part is probably why I am not rich, because I give them a lot of medication… and I’m losing a lot of money [on it]. My belief is: if you need it, even if you pay the R200 [USD15.00] consultation fee and the medication actually costs R300 [USD23.00], as long as you will be well [you get it]…”* (Male, age 30s)

Amidst these tensions, it was clear that while GPs sought financial profit, they aimed to minimise the burden to the client. Even though GPs described routinely referring clients with medical aid to private laboratories and specialists, giving them prescriptions to private pharmacies and recommending pricier brand-name drugs, their actions did not appear to incur any out-of-pocket expense to the user. Rather, they seemed to capitalise on steps they knew were covered by health insurance programs. Opting for prescriptions rather than direct

dispensation of drugs, for instance, also enabled GPs to facilitate insured clients greater agency in deciding on their preferred medicine brands at their subsequent pharmacy visit, or even when to procure them, in line with their pocket if they were paying for them in cash. When assessing TB risk, many GPs seemed to infer about or consider TB within their differential diagnosis, on the basis of their impression of the patient, that is, their physical appearance (whether kempt or unkempt, or state of clothing), the presenting history, and the medical insurance status. Given the epidemiology of TB globally and at national levels, and the increasing call to meet the needs of all affected communities including in particular those who are excluded or marginalised in various ways, these could indeed be defended as valid grounds for medical decision-making, rather than be seen as a form of race or class-based profiling.

> *"(**You mentioned that there are some symptoms; what symptoms do you look for**?) …. you going to be coughing, white sputum... if it's TB with cavities, there'll be blood. Then you're going to have a cough of more than 3 weeks, be losing weight, losing appetite, having night sweats, and have TB contacts. If someone you stay with is on TB treatment, usually the first two months are infective…. then obviously risk factors like HIV etc. … Poor socioeconomic status, uhm, living in shacks you know, so ja then you are exposed."* (Male, aged 40s)

Discussion

This study described the dynamics of antibiotic treatment for TB-like symptoms by private GPs. In general, GPs acknowledged that treating respiratory symptoms with broad-spectrum antibiotics was a common practice. The closer look into GP decisions accorded by this study reveals how TB diagnoses are potentially delayed in private GP settings. This may

be due to perceptions about clients and their expectations, and norms that deem empiric antibiotic treatment an acceptable and accessible way to bypass a lack of point-of-care tests for differential bacteriologic diagnoses including for TB. Indeed, from the providers' perspective, empirical antibiotic treatment seemed to constitute a systematic way of moving towards firm diagnoses, appropriate further investigations, and ultimately reduce the perceived burden of illness and the healthcare-seeking process on clients.

We have reported in a related analysis from the parent study that GPs in this setting know how TB typically presents (21). In the current study, however, some expressed concerns about other possible infections requiring immediate treatment. For example, symptoms that indicate "bad chest infections," "secondary bacterial infections," and infections that may spread to other members of the household appeared to warrant immediate antibiotic treatment. TB symptoms are generally nonspecific and overlap with those of other common bacterial respiratory infections, including *S. pneumoniae*, *S. aureus* and *H. influenzae* (38-40). In settings such as South Africa, a definitive TB diagnosis is further complicated by high HIV prevalence (41). Moreover, the high HIV prevalence coupled with the economic marginality of their clientele populations may warrant that GPs be vigilant regarding other common infections that could result in severe complications (42-44).

It is perhaps because of this context that GPs commonly reported using antibiotics as a means to rule out other differential diagnoses. Some referred to this as a systematic way to "treat basic things" before moving on to consider sputum tests and chest X-ray if there was no improvement. This sentiment seems to explain findings in the parent study involving standardised patient visits, where many GPs considered TB at the outset but asked clients to return to probe TB further if antibiotic treatment did not resolve the symptoms (21).

A seemingly grey area for GPs is the point at which antibiotics should be introduced for respiratory symptoms -- which could be TB-related -- and how (and when) the antibiotics' effectiveness or failure to draw a response should be determined. The 2020 South African Primary Healthcare Guidelines (45) recommend the use of amoxicillin or penicillin for suspected pneumonia in adults and children while also recommending simultaneous testing by Xpert Ultra to rule out TB disease in adults. In children, further investigation for TB is recommended in instances of severe pneumonia requiring hospitalisation, and where there is history of known TB contact, malnourishment or loss of weight, and when living with HIV (46).

As several GPs reiterated, they do not have the resources on hand to initiate tests for TB or other bacterial infections, which means that they send clients to private laboratories at a cost or refer to the public sector, which generally takes more time to navigate (47), for more in-depth evaluation. Thus, some even provided basic medications at low or no cost to the client, contrary to observations reported in other settings (48) that private providers commonly pursue profit-making through dispensing medicines. The practising environment in South Africa, as in settings such as the United States, further requires practitioners to hold licences to dispense medicines, and drug prices are fixed, leaving little room for physicians to profit from dispensing (49). For at least some GPs, it seems that treatment with antibiotics is the simplest way to avoid undue hardships and prevent possible complications for clients.

Yet, there are a number of problems with this approach. This not only can result in extended treatment delay and worsening health but can also be influenced by healthcare inequities. For some GPs, a client with health insurance may undergo more thorough examinations, including private sector tests, because they can afford them. In contrast, there

may be additional pressure on GPs to provide a “one stop shop” for those paying cash. Even if the effects are unintentional, deferring investigations into symptoms highly suggestive of TB in favour of promptly dispensing antibiotics may increase patient costs associated with transport, time, and the purchase of multiple medicines. In addition to the potential to mask symptoms, this can result in further health deterioration and severe and more advanced disease, and contribute to ongoing community transmission (50-53). The practice of profiling individuals on the basis of economic status to assess the TB risk level can further stigmatise people diagnosed with TB. In addition, GPs’ intent to rule out other potential infections, in this context of high HIV prevalence, might overlook the increasing use of antiretroviral treatment and growing rates of viral load suppression, which has reshaped the profile of infections in these populations.

Amid diagnostic resource constraints facing private general practice, GPs also appeared to grapple with applying their professional and experiential knowledge to process clinical presentations in ways that catered to users’ concerns and financial situation in these poorer communities. One result of these motley considerations is that GPs may give antibiotics, even as they may know the risk entailed (54, 55). Relatedly, some GPs also admitted to yielding to overt or implied client demands and expectations to receive antibiotics. Of note, a recent study of antibiotic use in South Africa reported that private providers worried that clients would not return if they did not receive antibiotics (56); however, other studies noted how providers may overestimate client expectations to receive medications (57-59).

Above all this, the perceived convenience of antibiotics to GPs, their reliance on experiential acumen, and their use of antibiotic treatment as a stopgap to simplify the

experience of clients at times negated the GPs' commitment to the overall wellbeing of clients and their own perceptions that they based decisions on clinical presentation and thorough history-taking. Effectively, rather than focusing on the common symptoms recommended nationally and globally (cough, fever, drenching night sweats, unintended weight loss), GPs seem to be triggered to investigate TB only after antibiotic treatment does not improve symptoms. Some even noted triggering investigations only in response to clients with a visibly deteriorated health state and/or prolonged duration of symptoms, in direct contrast with national and international guidelines.

In this study, a picture is presented where GPs and their practices (and management decisions for TB-related symptoms) are located in a complex milieu. The milieu potentiates contradictions, tensions, inconsistencies, and circumvention of norms. For example, GPs find themselves having to navigate complex and evolving national and international guidelines for TB and other respiratory illnesses, alternative diagnostic likelihoods, the inaccessibility of microbiological testing, and the complex social dynamics of the clients they serve (60). For some GPs, this results in deferring TB testing, placing clients (who in this instance are predominantly from marginalised socioeconomic status) at greater risk from severe TB outcomes as a result of diagnostic delay. Our findings mirror those of another study from India, which revealed key drivers of empirical therapy to include the common practice of using medications as diagnostic tools, the desire to provide rapid symptom relief to patients and manage their illness costs effectively, uncertainty about the presentation of TB, the effects of broad-spectrum antibiotics on TB symptomology, and uncertainty about the accuracy of available TB tests (53).

Conclusion

Optimising the patient-initiated pathway to TB diagnosis and treatment and enhancing the rational use of antibiotics in high-burden LMICs are important to lessen the disease burden and risk of drug resistance and toxicity (54, 61, 62). The quantitative outcomes of the parent study demonstrated that GPs have a good understanding of TB symptomatology and appropriate referral practices, but there are differences between their knowledge and observed practices (21). In this qualitative analysis, we illuminate what may underlie this 'know-do' gap, particularly in relation to the widespread empirical antibiotic treatment for symptoms suggestive of TB. Antibiotics appeared to be offered ahead of TB testing to treat or rule out some common infections, offer rapid symptom relief, and often as a means to prevent hardships from costly laboratory visits or the inconvenience of public sector testing for their clients, especially those deemed of lesser means. Some GPs may use it as a way to appease clients by giving them something tangible to take away. Ultimately, empirical antibiotic treatment for TB-like presentations in South Africa's private sector may best be explained by the absence of accessible and affordable TB and general bacteriologic tests initiated at the point of care. Additionally, given that the public sector in South Africa offers free GeneXpert and the providers' desire to mitigate hardship for this clientele population, one might argue that GPs have no easy way to refer their patients to the public sector for rapid diagnosis without the risk of losing track of them. Thus, it may be necessary to consider ways of making this referral easier while allowing GPs either to track the progress of, or to reconnect with, their clients following TB testing in the public sector.

As the WHO puts emphasis on achieving universal health care that is safe and of good quality, addressing inappropriate management practices will likely require action from

multiple fronts. This includes considering the complex context for GPs; supporting them in their desire to optimally serve patients, which could entail highlighting the implications of some aspects of their practices and decision-making; heightening GPs' suspicion index for TB or its prioritisation over diseases with similar symptoms in these contexts (21); and enabling GPs to navigate clients' pressures and expectations and the boundaries defining the latter's involvement in their own care. Perhaps most importantly, it will require diagnostic tools to be readily available and in close reach for primary care providers, including GPs. In part, this could mean connecting private practices to free tests through the public sector, as has been successfully demonstrated in pilot programmes in South Africa (63). South Africa has long recognised partnerships between the public and private sectors as a policy objective in health, and the national treasury has a unit that provides guidance on these partnerships. It has been argued, however, that stakeholders from both sectors need more mechanisms and forums for engaging in interactions in health and that contextual factors must inform the conception and management of these partnerships (64). Our study contributes to informing how public–private health engagement can be tailored and enhanced.

Among 212 GPs involved in the main study, we interviewed a small sample. While we achieved saturation on the major relevant themes, nuances based on gender and geography, among other factors, will r require more extensive research to discern. Interviews were also conducted during working hours, and some participants thus appeared hurried. Other participants thought the interviewers were performing an audit, despite the research assistants explicitly adopting a non-evaluative stance. These dynamics may have limited the depth of the interview conversations. It was also clear in some interviews that GPs who did not investigate TB during the standardised patient visits in the parent study assumed that

they had performed according to guidelines (note that individual-level performance was not disclosed to participants), so they spoke broadly about what they thought might lead to the practices of other GPs performing less well. This made it difficult at times to tease out, when GPs articulated decision-making processes, whether this was in relation to other GPs or their own thought processes and actions. Notwithstanding these overlaps, the narratives of GPs helped to draw out a vivid and complex picture of the context in which they practice, and offer explanations as well as opportunities for intervention to help achieve improved outcomes in TB.

Acknowledgements

The authors are grateful to the participants of this study. We would also like to thank Tsatsawani Mkhombo, Sean Jooste, Monalisa Jantjies, Linda Thumba, and Alicia North at the Human Sciences Research Council, South Africa, and Sarah Wu at McGill University Health Centre, Canada, for assisting with data collection and collation; Caroline Vadnais at McGill University Health Centre for assisting with research administration issues; the doctor associations for supporting the study in many ways. This work was supported in part by the Bill & Melinda Gates Foundation [Grant Number OPP1172634]. Under the grant conditions of the Foundation, a Creative Commons Attribution 4.0 Generic License has already been assigned to the Author Accepted Manuscript version that might arise from this submission.

Supplemental file

Semi-structured provider interviews

1. **Introduction and consent taking**
    a. Take a moment to remind them about the earlier study and that we want to learn a bit more about their experience to be able to contextualise the findings based on the simulated patient interactions.
    b. Try to get a sense of how much time they have for the interview.
    c. Explain that you are a social scientist and may not always be familiar with technical or clinical terms. Ask that they bear with you if you ask for clarification on some terminology.
2. **Let us talk a little about your practice**.
    a. Please describe to me the typical demographics of your patients.
    b. In an average week, what proportion of your patients are new (first-time)?
    c. What do you think brings patients back to your practice?
    d. What are some of the challenges you face with regard to having patients return to you?
    e. What makes you particularly proud of your practice?
    f. Please share with us/me what you enjoy about having a private practice in this community?
    g. Conversely what has frustrated you about having a private practice in this community.
        - If you could strengthen practice for GPs in <Durban/this community>, where would you start?

3. **We would like to learn a bit more about the typical complaints you see in your practice.**
    a. What are some of the most common conditions you encounter?
    (probe any conditions that may overlap with TB symptoms, e.g., flu, bronchitis, asking how doctor typically approaches treatment, what about patients who present with more general symptoms)
    What are some of the conditions you see that are more challenging to diagnose? Why is this the case?
    b. What conditions do you think are best serviced by the private sector? Why?
    c. Conversely, what conditions do you think are best treated in the public sector? Why?
4. **We'd now like to chat a bit about tests that you offer.**
    a. What are the common tests you usually offer patients on site? (does this differ between cash paying *vs medical aid patients)*
        i. If you send patients off site where do you usually send them to?
        ii. Are there specific tests that you would refer them to a public facility for? (Does this differ between cash paying and medical aid patients?)
    b. What about HIV testing
        i. How do you approach this subject with a patient?
        ii. Is there a different approach you have with a new patient vs a regular patient?
        iii. Do any other factors guide your approach such as the patient's gender?
        iv. How often do you see HIV in practice? (What proportion of your patients are positive)

5. **How about medications. Do you dispense medications yourself?**
    a. What are the common reasons for you to prescribe antibiotics – what are your favourite ones for respiratory conditions?
    b. What are the common reasons you would order a steroid?
    c. What about injections – what injections do you usually offer patients on site?
    d. We have heard that patients expect to leave a doctor's visit with medicines in hand – what is your opinion about this? How do you balance this with giving out potentially unnecessary medicines?
    e. On a typical day, what proportion of your patients would you prescribe medications to (probe: mostly all, seldom)?
6. **Let us talk about making decisions and acting with respect to presumptive TB.**
    a. How often do you see TB in your practice? (in the last 6 months how many people with TB did you see)
    b. What are some of the factors you consider before you suspect TB? (probe for auscultation)
    c. What are the challenges to diagnosing TB (*probe for generalised symptoms, more common diagnoses for these symptoms, complications involving HIV*)?
    d. What are the challenges to treating TB (probe referral to public sector, cash paying vs medical aid patients, and patient preferences)?
    e. There is a concept you may be familiar with called the TB cascade of care. The concept describes how a patient with TB may get lost in the healthcare system at various points before they are diagnosed or treated adequately. Part of the concern is the number of times a patient must make contact with the health

system, especially given that people with TB are more often of low socio-economic status. As a provider, how might you address this concern?

7 . Some of the work we do is around TB and HIV management. So we would like to round off by asking about your involvement in continuing medical education (CME), generally, or your or needs in this respect.

a. What are your CME needs specifically for TB-related diagnosis and management?/Are there particular aspects of TB that you would like to know more about? How about drug-resistant TB?

b. What are your CME needs regarding HIV diagnosis?

7. Is there anything else you would like to add?

Thank you very much for your time.

References

1. SABSSM VI Summary Sheet [press release]. Pretoria: HSRC, 2023 2023.
2. WHO. Global Tuberculosis Report. Geneva: World Health Organization; 2021.
3. WHO. Tuberculosis Profile: South Africa Geneva: World Health Organisation; 2024 [cited 2024 21 September]. Available from: https://worldhealthorg.shinyapps.io/tb_profiles/?_inputs_&entity_type=%22country%22&iso2=%22ZA%22&lan=%22EN%22.
4. WHO. Early detection of tuberculosis: an overview of approaches, guidelines and tools. Geneva: Author; 2011.
5. WHO. WHO consolidated guidelines on tuberculosis: Module 2: screening – systematic screening for tuberculosis disease. Geneva: Author; 2021.
6. WHO. WHO consolidated guidelines on tuberculosis. Module 4: treatment - drug-resistant tuberculosis treatment. Geneva: World Health Organisation; 2020.
7. WHO. WHO consolidated guidelines on tuberculosis. Module 5: management of tuberculosis in children and adolescents. Geneva: World Health Organization; 2022.
8. WHO. Automated real-time nucleic acid amplification technology for rapid and simultaneous detection of tuberculosis and rifampicin resistance: Xpert MTB. World Health Organization; 2013. Report No.: 9241506334.
9. WHO. Global tuberculosis report 2020. Geneva: Author; 2020.
10. DOH-SA. TB SCREENING AND TESTING: STANDARD OPERATING PROCEDURE. In: Health Do, editor. Pretoria: Department of Health South Africa; 2022.
11. WHO. Framework for conducting reviews of tuberculosis programmes. Geneva: Author; 2014.
12. WHO. Systematic screening for active tuberculosis: principles and recommendations. Geneva: Author; 2013. Report No.: 9241548606.
13. Ho J, Fox GJ, Marais BJ. Passive case finding for tuberculosis is not enough. International journal of mycobacteriology. 2016;5(4):374-8.
14. Hanson C, Osberg M, Brown J, Durham G, Chin DP. Finding the missing patients with tuberculosis: lessons learned from patient-pathway analyses in 5 countries. The Journal of infectious diseases. 2017;216(suppl_7):S686-S95.
15. Lehohla P. Use of health facilities and levels of selected health conditions in South Africa: Findings from the General Household Survey, 2011: Statistics South Africa Pretoria; 2013.
16. Econex. The South African Private Healthcare Sector: Role and Contribution to the Economy. Econex; 2013.
17. Christian CS, Gerdtham U-G, Hompashe D, Smith A, Burger R. Measuring quality gaps in TB screening in South Africa using standardised patient analysis. International journal of environmental research and public health. 2018;15(4):729.
18. Scott V, Azevedo V, Caldwell J. Improving access and quality of care in a TB control programme. South African Medical Journal. 2012;102(11):837-40.
19. Coarasa J, Das J, Gummerson E, Bitton A. A systematic tale of two differing reviews: evaluating the evidence on public and private sector quality of primary care in low and middle income countries. Globalization and health. 2017;13(1):1-7.
20. NDOH-SA. National Drug Policy for South Africa. In: Africa NDoHS, editor. Pretoria: NDoH-SA; 1996.
21. Boffa J, Moyo S, Chikovore J, Salomon A, Daniels B, Kwan AT, et al. Quality of care for tuberculosis and HIV in the private health sector: a cross-sectional, standardised patient study in South Africa. BMJ Global Health. 2021;6(5):e005250.

22. Salomon A, Boffa J, Moyo S, Chikovore J, Sulis G, Daniels B, et al. Prescribing practices for presumptive TB among private general practitioners in South Africa: a cross-sectional, standardised patient study. BMJ Global Health. 2022;7(1):e007456.
23. DOH. Adherence guidelines for HIV, TB and NCDS. Policy and service delivery guidelines for linkage to care, adherence to treatment and retention in care. In: Health Do, editor. Pretoria: author; 2016.
24. DOH. National Tuberculosis Management Guidelines. In: Health Do, editor. Pretoria: author; 2014.
25. Benoot C, Hannes K, Bilsen J. The use of purposeful sampling in a qualitative evidence synthesis: A worked example on sexual adjustment to a cancer trajectory. BMC Medical Research Methodology. 2016;16(1):21.
26. Saunders B, Sim J, Kingstone T, Baker S, Waterfield J, Bartlam B, et al. Saturation in qualitative research: exploring its conceptualization and operationalization. Quality & quantity. 2018;52:1893-907.
27. Kvale S. Interviews: An introduction to qualitative research interviewing: Sage Publications, Inc; 1994.
28. Lincoln Y, Guba E. Naturalistic inquiry1985.
29. Coffey A, Atkinson P. Making sense of qualitative data: Complementary research strategies: Sage Publications, Inc; 1996.
30. Armat MR, Assarroudi A, Rad M, Sharifi H, Heydari A. Inductive and Deductive: Ambiguous Labels in Qualitative Content Analysis. The Qualitative Report. 2018;23(1):219-21.
31. Charmarz K. The search for meaning grounded theory. In: Smith JA, Harre´ R, van Langenhove L, editors. Rethinking methods in psychology London: Sage; 1995. p. pp. 27-65.
32. Glaser BG. Emergency vs. reinforcing: Basics of grounded theory analysis. Mill Valley: Sociology Press; 1992.
33. Glaser BG, Strauss AL. The discovery of grounded theory: Strategies for qualitative research: Transaction Books; 2009.
34. Thornberg R, Charmaz K. Grounded theory and theoretical coding. The SAGE handbook of qualitative data analysis. 2014;5:153-69.
35. Hinds PS, Chaves DE, Cypess SM. Context as a source of meaning and understanding. Qualitative health research. 1992;2(1):61-74.
36. Tong A, Sainsbury P, Craig J. Consolidated criteria for reporting qualitative research (COREQ): a 32-item checklist for interviews and focus groups. International Journal for Quality in Health Care. 2007;19(6):349-57.
37. Olatunbosun OD, Esterhuizen TM, Wiysonge CS. A cross sectional survey to evaluate knowledge, attitudes and practices regarding seasonal influenza and influenza vaccination among diabetics in Pretoria, South Africa. Vaccine. 2017;35(47):6375-86.
38. Feldman C, Brink A, Bateman GA, Maartens G, Bateman ED. Management of Community-acquired Pneumonia in Adults. South African Medical Journal. 2007;97(12):1295-306.
39. STATS-SA. Mortality and causes of death in South Africa, 2009: Findings from death notification. In: Africa SS, editor. Pretoria: Statistics South Africa; 2011.
40. REACH. National syndromic surveillance for pneumonia in South Africa Cape Town: Research Centre for Adolescent and Child Health; n.d. [cited 2024 14 October]. Available from: https://health.uct.ac.za/department-paediatrics/research-about-research-department-research-units-research-centre-adolescent-and-child-health-reach/national-syndromic-surveillance-pneumonia-south-africa.
41. World Health Organization. Global tuberculosis report 2020. 2020.
42. Theron G, Peter J, Dowdy D, Langley I, Squire SB, Dheda K. Do high rates of empirical treatment undermine the potential effect of new diagnostic tests for tuberculosis in high-burden settings? The Lancet infectious diseases. 2014;14(6):527-32.

43. Lawn S, Ayles H, Egwaga S, Williams B, Mukadi Y, Santos Filho E, et al. Potential utility of empirical tuberculosis treatment for HIV-infected patients with advanced immunodeficiency in high TB-HIV burden settings [Unresolved issues]. The International journal of tuberculosis and lung disease. 2011;15(3):287-95.
44. Bresges C, Wilson D, Fielding K, Corbett EL, Del-Greco F, Grint D, et al., editors. Early empirical TB treatment in HIV-positive patients admitted to hospital in South Africa: an observational cohort study. Open Forum Infectious Diseases; 2021: Oxford University Press (OUP).
45. Health TNDo. Primary Healthcare Standard Treatment Guideline and Essential Medicine List. 7th ed ed. Pretoria: The National Department of Health, South Africa: Essential Drugs Programme.; 2020.
46. Zar HJ, Moore D, Itzikowitz G, Green RJ, Argent A, Avenant T, et al. Diagnosis of community-acquired pneumonia in children: South African Thoracic Society guidelines (part 2). South African Medical Journal. 2020;110(7):588-93.
47. Govender K, Girdwood S, Letswalo D, Long L, Meyer-Rath G, Miot J. Primary healthcare seeking behaviour of low-income patients across the public and private health sectors in South Africa. BMC Public Health. 2021;21:1-10.
48. Kwan A, Daniels B, Saria V, Satyanarayana S, Subbaraman R, McDowell A, et al. Variations in the quality of tuberculosis care in urban India: a cross-sectional, standardized patient study in two cities. PLoS medicine. 2018;15(9):e1002653.
49. Act No. of 201 : Medicines and 14 5 Related Substances Amendment Act, 2015, 39585 (2015).
50. Craig S, Bettinson H, Sabin C, Gillespie S, Lipman M. Think TB! Is the diagnosis of pulmonary tuberculosis delayed by the use of antibiotics? The International Journal of Tuberculosis and Lung Disease. 2009;13(2):208-13.
51. Wang M, Fitzgerald J, Richardson K, Marra C, Cook V, Hajek J, et al. Is the delay in diagnosis of pulmonary tuberculosis related to exposure to fluoroquinolones or any antibiotic? The International journal of tuberculosis and lung disease. 2011;15(8):1062-8.
52. Wang J-Y, Hsueh P-R, Jan I-S, Lee L-N, Liaw Y-S, Yang P-C, et al. Empirical treatment with a fluoroquinolone delays the treatment for tuberculosis and is associated with a poor prognosis in endemic areas. Thorax. 2006;61(10):903-8.
53. McDowell A, Pai M. Treatment as diagnosis and diagnosis as treatment: empirical management of presumptive tuberculosis in India. The International Journal of Tuberculosis and Lung Disease. 2016;20(4):536-43.
54. Dixon J, Manyau S, Kandiye F, Kranzer K, Chandler CIR. Antibiotics, rational drug use and the architecture of global health in Zimbabwe. Social Science & Medicine. 2021;272:113594.
55. Broniatowski DA, Klein EY, May L, Martinez EM, Ware C, Reyna VF. Patients' and Clinicians' Perceptions of Antibiotic Prescribing for Upper Respiratory Infections in the Acute Care Setting. Med Decis Making. 2018;38(5):547-61.
56. Lagarde M, Blaauw D. Levels and determinants of overprescribing of antibiotics in the public and private primary care sectors in South Africa. BMJ Global Health. 2023;8(7):e012374.
57. May L, Gudger G, Armstrong P, Brooks G, Hinds P, Bhat R, et al. Multisite exploration of clinical decision making for antibiotic use by emergency medicine providers using quantitative and qualitative methods. Infect Control Hosp Epidemiol. 2014;35(9):1114-25.
58. Welschen I, Kuyvenhoven M, Hoes A, Verheij T. Antibiotics for acute respiratory tract symptoms: patients' expectations, GPs' management and patient satisfaction. Family Practice. 2004;21(3):234-7.
59. Lado E, Vacariza M, Fernández-González C, Gestal-Otero JJ, Figueiras A. Influence exerted on drug prescribing by patients’ attitudes and expectations and by doctors’ perception of such expectations: a cohort and nested case-control study. Journal of Evaluation in Clinical Practice. 2008;14(3):453-9.

60. Houben RMGJ, Esmail H, Cobelens F, Williams CML, Coussens AK. Tuberculosis prevalence: beyond the tip of the iceberg. The Lancet Respiratory Medicine. 2022;10(6):537-9.
61. WHO. Systematic screening for active tuberculosis: an operational guide. Geneva: World Health Organization; 2015. Geneva: WHO/HTM/TB; 2015.
62. Bresges C, Wilson D, Fielding K, Corbett EL, Del-Greco F, Grint D, et al., editors. Early empirical tuberculosis treatment in HIV-positive patients admitted to hospital in South Africa: an observational cohort study. Open forum infectious diseases; 2021: Oxford University Press US.
63. Chibi B SS-HK, Moyo S, Naidoo M, Mhlaba T, Mapham W, Lutchminarain K, Chikovore J, Boffa J. Feasibility of linking clients presenting with TB symptoms in South Africa's private sector to timely testing and treatment in public sector: Connect TB Study [abstract]. Int J Tuberc Lung Dis. 2022;26(Supp 2):(S106).
64. Kula N, Fryatt RJ. Public-private interactions on health in South Africa: opportunities for scaling up. Health Policy Plan. 2014;29(5):560-9.

Click here to access/download;Figure;Figure1_Chikovore_23Sep2024.docx

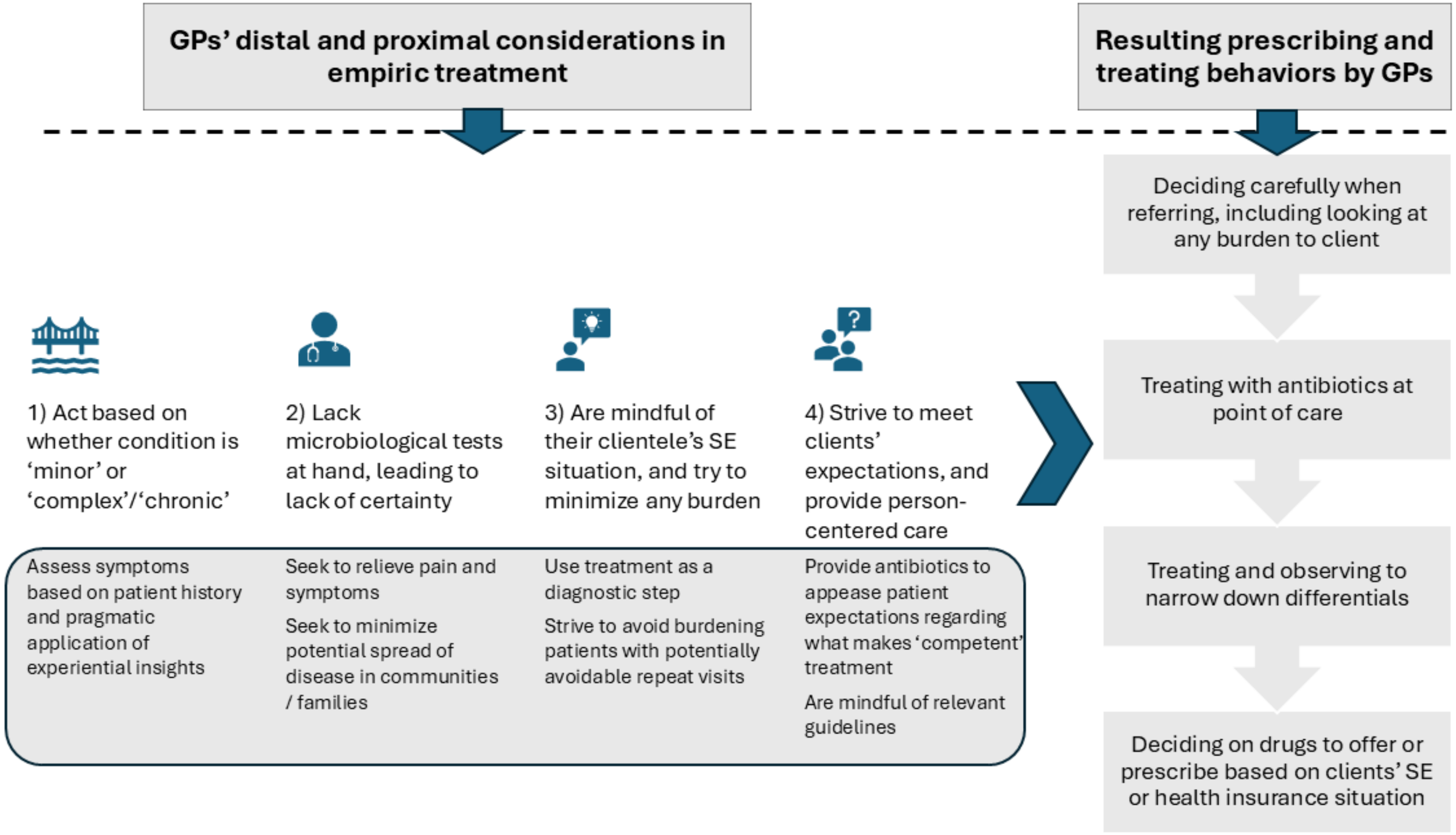

SE = Socioeconomic; GP = General practitioner

Fig 1. Private general practitioners' antibiotic treatment practices and decision-making considerations
