# Supplementary material for: Provider perspectives on empirical antibiotic treatment for tuberculosis-like symptoms in South Africa’s private general practice sector: A qualitative study in two cities
Source: PLOS Glob Public Health. 2025 Jun 24;5(6):e0004742. doi: 10.1371/journal.pgph.0004742 (PMC12186942; doi:10.1371/journal.pgph.0004742)
Supplement: Attachment — Submitted filename: Response to reviewers.docx [file pgph.0004742.s005.docx]

**MANUSCRIPT PGPH-D-24-02250: Reviewer comments and responses from the authors**

**Journal requirements**:

| Comment | Response |
| --- | --- |
| 1. Please include a complete copy of PLOS’ questionnaire on inclusivity in global research in your revised manuscript. Our policy for research in this area aims to improve transparency in the reporting of research performed outside of researchers’ own country or community. The policy applies to researchers who have travelled to a different country to conduct research, research with Indigenous populations or their lands, and research on cultural artefacts. The questionnaire can also be requested at the journal’s discretion for any other submissions, even if these conditions are not met. Please find more information on the policy and a link to download a blank copy of the questionnaire here: [https://journals.plos.org/globalpublichealth/s/best-practices-in-research-reporting](https://url.za.m.mimecastprotect.com/s/umMpCP1Jv9fvL8lvH0h0HxrM3A). Please upload a completed version of your questionnaire as Supporting Information when you resubmit your manuscript. | The questionnaire has been downloaded, to be completed and submitted as a supplemental file. |
| 2. Please provide separate figure files in .tif or .eps format. | The figure has been saved in tiff format. |

**Editor’s comments**

We are very thankful for these positive sentiments conveyed regarding the significance of our study.

**Reviewer #1 comments**

| Comment | Response |
| --- | --- |
| First of all, thank you for submitting this important manuscript on elucidating the provider perspective on empirical antibiotic treatment for tuberculosis-like symptoms. The timing of this manuscript is pivotal due to complexities and trade off the health provider needed to make to serve their patients while maintaining objectivity to the guideline. I have spotted the places where comments are required to addressed in the form of the comment. However, some of the comments I understand will require mentioning here. | We appreciate these positive sentiments. |
| A. In the abstract, you have talked about your participant selection criteria on 15 per site. I am not clear about the rationale of doing so. Even though they are two urban cities, it is imperative to have some of the attributes regarding the type of patients they have seen, their specialities. Age is an important parameter need more elaborate the diversification of the age group to understand the perception of young and the old healthcare providers. | We thank you for these important queries. With regard to sample size rationale, with a view to achieving saturation, and balance feasibility of undertaking a qualitative supplementary study, we decided on a sample of 30 overall, purposively including GPs who perfumed well and not well (lines 139-140). We believe we achieved saturation on this main analytic axis. We specify that this is a small sample and more intricate comparative analyses - such as by GP age or gender, etc.- were not possible, in Limitations/Discussion.  With regard to attributes, by definition, GPs are general and do not have specialisation. Moreover, we approached GPs whose clientele included adults, and we describe the common ailments these GPs collectively saw (lines 205-214). Specifying details by individual GP may breach confidentiality. Rather we have tabulated socio demographic and practice characteristics in Table 1. |
| B. You have given a glimpse on the management decision regarding the expectation of referral or the demand regarding on the spot treatment. Can you elaborate on which type of disease seen by these groups of GPs faced more demand from the patient regarding on the spot treatment. | We thank you for this comment. GPs saw a wide variety of ailments/diseases as stated above but identifying an association between the type of disease seen vs patient demand was beyond the scope of this qualitative study. |
| C. Please provide some info on the multi-drug resistant TB being different and relevant to the South African context from other countries of TB. Since the paper is on TB-like symptoms, please kindly provide the data on the prevalence of TB-like symptoms and the causative pathogen that can be linked to these symptoms. | Thank you for this suggestion. We have revised the first paragraph of the Introduction to highlight the burden of drug-resistant TB (lines 56-62).  With regard to providing data on the prevalence of TB-like symptoms, we have referred to The First National TB Prevalence Survey in South Africa, carried out in 2018, which established that 14.7% of survey participants reported TB symptoms (lines 86-87).  Regarding the causative agent: TB shares symptoms with various other lung conditions or health problems and thus it is not feasible to link to a causative pathogen on the basis of symptoms only. We describe the common symptoms associated with TB in lines 424-425 and acknowledge these symptoms can be indicative of other pulmonary conditions in lines 425-426. |
| D. In line 70, please add information of the TB diagnostic coverage to understand the context better | We have inserted in line 69-70 the following text “was rolled out in 2017 and by 2019, 203 testing sites were servicing 4 710 facilities” |
| E. The introduction also require some justification focusing on the need of private healthcare infrastructure. What are the failures of the public health infrastructure. It is unfortunately not clearly articulated. Regarding the data provided for the private healthcare services, how much it is attributable to the infectious diseases and how much to chronic non-communicable disease. | Thank you for pointing to this oversight. We have reviewed and edited extensively throughout the introduction to address justification for focusing on the private GPs sector (lines 84-125).  We have specified now which statements on private care relate to any illness (eg lines 89-91) vs just TB (lines 93-99). A comparison between NCDs and IDs has not been performed to our knowledge. |
| F. Please kindly highlight the gaps and impact of empirical antibiotic usage and why it remains a large scope of a problem in the South African landscape in terms of drug resistance, supply chain of antibiotics regarding TB-like symptoms. | In lines 119-123, we now describe the extent of antibiotic treatment by GPs within the parent study. We further revisit the impact of antibiotic use and resistance in detail in the discussion (lines 411-418; line 456-475; lines 486-495; lines 501-507) . |
| G. In your participant enrolment, I have seen a higher dominance of male in both site. Can you explain a bit how much it is similar in terms of gender parity in private healthcare providers (Male-to-Female Ratio). If it is widely different, why selecting 75% male participants. | We thank you for this query. An estimated 60% of private GPs are male in South Africa (see https://www.researchgate.net/figure/Breakdown-of-registered-doctors-by-population-group-age-and-sex-South-Africa-2019_fig5_349730722). While the GP gender ratio in areas with high TB notification rates, where we based our parent study, is not known, we did anticipate a higher number of male GPs.  We have clarified in Table 1 that our sample comprised 83.3% male GPs, and under Limitations/Discussion, that we were unable to make gender comparisons, as nuances based gender will require more extensive research to discern (lines 542-544).  Kindly also refer to our response to Query ‘A’ above, as these are related. |
| H. In the results, you have talked about the key insights with respect to providing quotes. Can you give a brief how common was each of the scenarios you have identified. Even though its a qualitative study, a distribution of the themes can be illustrated. The best can be described in the results with a brief summary of the themes from the 30 participants which will give a certain importance of the findings identified through this study. As it currently, these insights are unique and only talked about by the individual healthcare provider. | We thank you for this comment. We have summarized the main themes and key points within each theme in Table 2.  We appreciate the suggestion to present a distribution of themes and acknowledge that some qualitative studies do present themes in this way. Variation in how results are presented arises from variations in methodologies and theoretical approaches in qualitative research. The decision to count, often the exception in these studies, is pragmatically made and germane to the study’s goals, design, assumptions, and even target audience. Our approach and incline lean against quantifying, and we prefer to dwell on each incident in detail and contextualize it, against other incidents but also in the immediate and broader context of the study. This approach reflects in the ways we pay attention to different sub-themes and also bring them all together while teasing them out in the discussion.  A note that our themes were generated after data from different sources (participants) were read and combined ‘fittingly’; hence, themes were not drawn from individuals interviews and then displayed. Rather, they represent a whole constructed from bits and slices picked from individual interviewees. They are intentionally high level, meaning that it took reading the entire interview or a very large chunk of it to see how a participant contributed to that theme profiled. All of this happened with a great deal of critical reflexivity and qualitative rigour checks on the part of our team. We detail or analytic approach in quite some detail in the Methods/Analysis section (lines 165-193). |
| I. The scenarios and insights that has been depicted are plausible. However, they may be skewed to particular disease profile of the patients. Can you stratify the trade off on the antibiotic prescribing by patient profile which will be helpful to understand the insights better. | Thank you for this comment. We have understood this to suggest there could be justification for empirical treatment based on the profile of a patient. Indeed, treatment was said to depend on patient profile (including socioeconomic factors, patient history, presenting symptoms, and how the provider interpreted available information using their experiential insights). We therefore agree that disease profile is factored in, although it emerged to not (always) be the only consideration. We thus cannot stratify on this basis alone.  In relation to findings being skewed towards a certain disease profile: This qualitative study indeed focused on management of TB-like symptoms, and to illuminate the nuance in varied prescribing practices. The parent study revealed widespread antibiotic use, notable know-do gaps, and GPs not referring for or inquiring about things such as HIV (lines 106- 125). The entire study suite thus addressed the management of TB-like symptoms, with the qualitative sub-study seeking to enhance insights into the contextual dynamics of antibiotic use by the GPs, specifically in the care provision for people with TB-like symptoms (123-125). This background is therefore elaborated in the introduction. |
| J. Furthermore, the insights you have shared can you differentiate on the characteristics you have depicted regarding location, years in practice (young vs old), higher patient load and consultation fees to understand the impact on the decision making regarding empirical treatment. | We thank you for this query. (We wish to also refer to our related response to ‘G’ and ‘H’ above).  Our sample size did not allow us to ascertain any trends according to demographic characteristics. As we have indicated in response to some queries above (including about the GP sample), the nature of the research, he sample size, and the specific approach we took, do not permit identifying a theme and determining its associations. This also has to do with themes being complex forms, for which it is difficult to quantify or establish trends.  This notwithstanding, we present themes and illustrative quotes, with only limited demographics to maintain anonymity and confidentiality of the GPs, and the participants whom we quote tend to vary in age. However, it is, as we highlight above, not feasible to analyze trends by age. |
| K. Can you highlight the scopes and threats of indiscriminate prescribing with respect to your findings. | We thank you for this comment. We wish to draw attention also to our related response to ‘F’ above, on empirical antibiotic usage and why it remains a large scope of a problem in the South Africa.  Indiscriminate prescribing (which we read as related to empirical treatment) was common as described by GPs throughout the findings in this qualitative study, reiterating findings of the parent study. It can delay diagnosis, prolong illness potentially leading to death, increase care-seeking costs, and drive community spread and even DR-TB. We have highlighted and reiterated these threats at different points in the discussion and conclusion (lines 411-418; line 456-475; lines 486-495; lines 501-507) |
| L. Please kindly highlight in the discussion in lights of the results with regards to HIV problem. How much patients with HIV are being seens in these private practices | HIV is an important consideration in the TB public health domain, and equally for South Africa, and guidelines for managing and diagnosing TB also incorporate HIV. In our study (both the parent and qualitative studies), we thus evaluated GPs’ consideration of and inquiry about HIV for their patients presenting with TB-like symptoms. The syndemic context of TB/HIV means that both private providers and the public sector likely see and manage people with HIV, or both TB and HIV. We also mention in different parts of the discussion the relevance of HIV considerations (for example, lines 427-430; lines 442-445; lines 469-475; lines 568-570) |
| Additional comments from Reviewer #1 | We have also addressed the comments included directly in the manuscripts pdf through edits throughout the revised version. The following from the pdf may not have been previously addressed. |
| (pdf comment) Did the testing coverage considered | We are thankful for this comment. The entire study (including the parent study - which has been published) sought to document practices of GPs in relation to managing TB-related symptoms. From the published findings (which are not repeated in this paper), ideal management (which was defined as being recommended a test for TB or HIV or being referred to the public sector for any reason) occurred in 57% of the interactions with symptomatic TB actor patients. Testing alone was not computed. |
| clarify if it applies for someone having clinical presentation with co morbidities | We thank you for this query. In light of descriptions given throughout the findings, GPs certainly considered the presenting history of the patients, and their presenting symptoms when making the call to treat or refer |
| study limitation could be added | We thank you for this suggestion. A limitations section has now been specifically designated (lines 533-579). |
| Discussion should compare with the relevant literatures with explaining gp picture regional  developed country comparison. / | Thank you for this guidance. We have referred to literature in the discussion and conclusion (lines 427-430; lines 448-455; line465; lines 474-7; lines 480-485, lines 501-507; lines 510-516; lines 568-570; and lines 595-599) |

**Reviewer #2 Comments**

| Comment | Response |
| --- | --- |
| This is an insightful study on how private general practitioners in South Africa navigate diagnostic challenges and socioeconomic pressures when managing TB-like symptoms, often relying on empirical antibiotics that delay TB diagnosis. Drawing on qualitative insights from 30 practitioners, it identifies key barriers and offers actionable recommendations to improve TB care and antibiotic stewardship. I commend the authors for their nuanced approach to this critical public health issue and for providing meaningful solutions to enhance TB management in resource-limited, high-burden settings. I would like to suggest some points to improve the manuscript further | We thank you for these positive sentiments. |
| Abstract: It would be helpful to highlight the most impactful results (e.g., how empirical antibiotic treatment delays TB diagnoses) I suggest that the authors emphasise actionable recommendations, such as integrating affordable TB tests in private practices. To improve conciseness, I suggest reducing the length by removing less critical background details (e.g., TB burden statistics). | We thank you for the suggestion. We have edited the abstract, and incorporated, in addition to making it more concise. However, since there are no affordable tests in existence, we have phrased thus: “linking GPs directly to public sector TB tests for clients” |
| Introduction: Please consider streamlining the introduction section. The introduction is comprehensive but could be streamlined for example shifting specific details (e.g., diagnostic statistics) to the discussion or methods section. For the statistics, only include essential data to set the context. Please consider highlighting empirical antibiotic use as the issue being addressed. Introduce the research gap upfront instead of elaborating extensively on TB burden or healthcare system shortcomings. The authors could consider presenting the study's aim in a single, concise sentence with a focus on its novelty and contribution. | We thank you for the suggestion. In balancing the critical need to streamline, and the request for additional detail from Reviewer #1, we have rearranged text and also removed some in various areas of the introduction. Regarding bringing empirical treatment upfront, we acknowledge that this is the focus of the paper. However, bringing it upfront might move it out of the wider crucial context of TB, and more specifically, of private practice usage, the gaps therein, and the specific context (of the larger study) in which we then ended up focusing empirical treatment. |
| Methods |  |
| 1) Design & participants – Please specify any criteria for GP selection beyond variation in age, sex, and practice location. For example, were there thresholds for years of practice or experience in treating TB/HIV cases? While 30 participants were chosen to achieve thematic saturation, provide a more explicit justification or reference similar studies with comparable sample sizes. | Please refer to our response to ‘A’ from Reviewer #1. |
| 2) Data collection – Please elaborate on steps taken to mitigate potential interviewer bias, particularly since participants were aware of being part of a TB study. I would be wise to include some explanation (potentially in the discussion section) of how conducting interviews during GP working hours might have influenced responses and suggest alternatives for future studies. | We appreciate this comment. We managed interviewer bias by asking open ended non evaluative questions, etc. (see the interview guide, Supplemental file). While participants were aware that this was a TB study, their candid responses actually suggest they were very open to sharing their decisions and rationale and did not feel inhibited from revealing insights that deviated from best practice (as is evident from many quotes). Hence we are confident interviewer bias was overcome. We also followed COREQ Guidelines to help ensure rigour in our process.  We acknowledge that interviews during working hours might have influenced responses, and we have addressed this in lines 545-554) under the limitations. |
| 3) Data analysis – Please expand on how the coding framework was developed, particularly for the "large chunks" of data. Was it based solely on objectives, or were external coding schemes used? Authors may want to provide a more detailed explanation of how confirmatory or alternate perspectives from the research team influenced theme refinement. Please address any potential concerns about the "audit perception" by participants and how this was mitigated. For ethical purposes, authors may want to provide more specifics (i.e. strategies) on how participant confidentiality was ensured beyond using anonymized labels for quotes. | We thank you for this query. The data were initially coded in large chunks on the basis of the study objectives and preliminary analysis of the larger study; not (initially) solely based on study objectives). We have added text providing more detail in the analysis section lines(lines 175-184). We have also added text regarding how confirmatory or alternate perspectives from the research team influenced theme refinement, in the Data management and analysis section (lies 185-191). We have also added text that clarifies the concern regarding the "audit perception" by GPs participants and how this was mitigated, in limitations section (lines 546-554). In addition, we have provided additional details under the sections on Data Management and analysis, on how participant confidentiality was ensured beyond using anonymized labels for quotes (lines 168-173). |
| 4) Rigour of the study- Please explain strategies to ensure rigour of the study (i.e. principles by Lincoln & Guba) | We thank you for this comment. We have added new text in the tracked version (Lines 571-579). In addition, as highlighted above, we also followed COREQ Guidelines to help ensure rigour in our process. |
| 5) Results: Please include a discussion on the skewed gender distribution (25 males, 5 females) and its potential impact on findings. To improve readability, please consider organising the findings under clearly defined subheadings. For example 1) antibiotic prescription practices; 2) perceived client pressure; 3) socioeconomic considerations. If this is within the limit, consider including figures or tables for a summary of themes or decision-making factors among GPs for better readability. I suggest shortening participant quotes where possible and ensuring they directly support the narrative. | The gender ratio of interviewed GPs has been addressed under Query G from Reviewer #1. Regarding the impact on the findings, we acknowledge the sample size being a limitation (Lines 542-544). We have organized the findings as suggested, and this was really helpful. We have now included a table of the summary of themes (Table 2). We have reviewed some quotes, the extent possible shortening them, while ensuring the essence of their meaning is retained. |
| 6) Discussion: The discussion should focus on integrating study findings with existing knowledge, addressing implications for policy and practice, and identifying avenues for further research. Please consider my comments for the discussion section as mentioned above. I would suggest the authors consider adding subheadings on these aspects: 1) Key summary of the findings 2) Strengths & limitations of the study 3) Comparison of study findings to global evidence 4) Implications for policy, practice & future research. | We are grateful for these suggestions, and we have reorganized the discussion section. Regarding comparing study findings to global evidence, please see our response Reviewer #1’s last query above. |
