# Supplementary material for: Provider perspectives on empirical antibiotic treatment for tuberculosis-like symptoms in South Africa’s private general practice sector: A qualitative study in two cities
Source: PLOS Glob Public Health. 2025 Jun 24;5(6):e0004742. doi: 10.1371/journal.pgph.0004742 (PMC12186942; doi:10.1371/journal.pgph.0004742)
Supplement: Attachment — Submitted filename: Response_to_reviewers_auresp_2.docx [file pgph.0004742.s006.docx]

18 April 2025

Dr Catherine Kyobutungi and Professor Madhukar Pai

Editors-in-Chief

PLOS Global Public Health

200- 1265 Battery Street

San Francisco, CA 94111

**Re: Re-Submission of research article, PGPH-D-24-02250 - “Provider perspectives on empirical antibiotic treatment for tuberculosis-like symptoms in South Africa’s private general practice sector: A qualitative study in two cities”**

Dear Dr Kyobutungi and Professor Pai,

We would like to thank you and your reviewers for the tremendously helpful feedback that assisted us to revise and improve our manuscript. We have revised the manuscript, and we believe we have attended to all the comments and suggestions to your satisfaction.

Please kindly accept our revised manuscript.

Below we detail the revisions that we have implemented, and also provide responses to the queries raised.

**Journal requirements**:

| Comment | Response |
| --- | --- |
| 1. 1. Please review your reference list to ensure that it is complete and correct. If you have cited papers that have been retracted, please include the rationale for doing so in the manuscript text, or remove these references and replace them with relevant current references. Any changes to the reference list should be mentioned in the rebuttal letter that accompanies your revised manuscript. If you need to cite a retracted article, indicate the article’s retracted status in the References list and also include a citation and full reference for the retraction notice. | We thank you for bringing this to our attention. We have reviewed references to ensure that they are complete. We made the following specific changes:   - References 3 and 4: We replaced “Author” with “World Health Organisation”. - Reference 9: We edited from “In: Health Do, editor. Pretoria: author; 2014” to become “Pretoria: Department of Health; 2014” - Reference 10: We edited from “DOH-SA. TB SCREENING AND TESTING: STANDARD OPERATING PROCEDURE. In: Health Do, editor. Pretoria: Department of Health South Africa; 2022” to become “DOH-SA. TB Screening and Testing: Standard Operating Procedure. Pretoria: Department of Health; 2023.” - Reference 15: We changed “Geneva: Author; 2014” to become “Geneva: World Health Organisation; 2014” - Reference 16: We replaced “Author” with “World Health Organisation” - Reference 27: We edited from “Government SA. NATIONAL TB RECOVERY PLAN 3.0: April 2024 – MARCH 2025. In: Health Do, editor. Pretoria: DoH; 2024. To become “SAG. National TB Recovery Plan 3.0: April 2024 – MARCH 2025. Pretoria: South African Government; 2024” - Reference 31: We edited from “NDOH-SA. National Drug Policy for South Africa. In: Africa NDoHS, editor. Pretoria: NDoH-SA; 1996” to become “DOH-SA. National Drug Policy for South Africa. Pretoria: Department of Health South Africa; 1996”. - Reference 35: We edited from “DOH. Adherence guidelines for HIV, TB and NCDS. Policy and service delivery guidelines for linkage to care, adherence to treatment and retention in care. In: DOH-SA, editor. Pretoria: author; 2016.” to become “DOH-SA. Adherence guidelines for HIV, TB and NCDS. Policy and service delivery guidelines for linkage to care, adherence to treatment and retention in care. Pretoria: Department of Health South Africa; 2016.” - Reference 51: We added city “Geneva” - Reference 53: We edited from “STATS-SA. Mortality and causes of death in South Africa, 2009: Findings from death notification. In: Africa SS, editor. Pretoria: Statistics South Africa; 2011” to become “STATS-SA. Mortality and causes of death in South Africa, 2009: Findings from death notification. Pretoria: Statistics South Africa; 2011.” - Reference 55” We replaced this the updated version, “WHO. Global Tuberculosis Report 2024. Geneva: World Health Organization; 2024.” - The existing reference 58, “Health TNDo. Primary Healthcare Standard Treatment Guideline and Essential Medicine List. 7th ed ed. Pretoria: The National Department of Health, South Africa: Essential Drugs Programme; 2020.” Was renumbered to 58, and edited to become “NDOH-SA. Essential Drugs Programme. Primary Healthcare Standard Treatment Guideline and Essential Medicine List. 7th ed. Pretoria: The National Department of Health South Africa; 2020.” - Existing reference 63 “Act No. of 201 : Medicines and 14 5 Related Substances Amendment Act, 2015, 39585 (2015).” Was renumbered to 62 and edited to become “SAG. Medicines and Related Substances Amendment Act 14 of 2015. Pretoria: South African Government; 2015” |

**Reviewers' comments:**

**Comments to the Author
Reviewer #2 comments**

| Comment | Response |
| --- | --- |
| Table 1 effectively presents provider characteristics; however, the categorisation of "Approximates age" could be more structured and presented in a more orderly manner for improved clarity. | We thank you for this very helpful comment. We have reordered the presentation of our participants’ approximate ages, by presenting more clearly according to the ages, for the overall sample of 30 participants and also according to city. |
| While the gender imbalance is mentioned in the Discussion, its potential impact on findings has not been critically analysed. | We thank you for this query. We understood this query (together with others related to it) as having been raised in earlier reviews. In our previous responses, we acknowledged that the gender imbalance was a potential limitation, and furthermore, noted that we could not discern nuances by gender and geography as these would require more extensive research to ascertain.  Below, we reshare the previous comments and the responses that we gave in full. We are hopeful that our current response, together with previous responses, are sufficient to address the reviewer’s query. |
| Previous query  *A. In the abstract, you have talked about your participant selection criteria on 15 per site. I am not clear about the rationale of doing so. Even though they are two urban cities, it is imperative to have some of the attributes regarding the type of patients they have seen, their specialities. Age is an important parameter need more elaborate the diversification of the age group to understand the perception of young and the old healthcare providers.* | Our previous response  *We thank you for these important queries. With regard to sample size rationale, with a view to achieving saturation, and balance feasibility of undertaking a qualitative supplementary study, we decided on a sample of 30 overall, purposively including GPs who perfumed well and not well (lines 139-140). We believe we achieved saturation on this main analytic axis. We specify that this is a small sample and more intricate comparative analyses - such as by GP age or gender, etc.- were not possible, in Limitations/Discussion.* |
| Previous query  *G. In your participant enrolment, I have seen a higher dominance of male in both site. Can you explain a bit how much it is similar in terms of gender parity in private healthcare providers (Male-to-Female Ratio). If it is widely different, why selecting 75% male participants.* | Our previous response  *We thank you for this query. An estimated 60% of private GPs are male in South Africa (see https://www.researchgate.net/figure/Breakdown-of-registered-doctors-by-population-group-age-and-sex-South-Africa-2019_fig5_349730722). While the GP gender ratio in areas with high TB notification rates, where we based our parent study, is not known, we did anticipate a higher number of male GPs.*  *We have clarified in Table 1 that our sample comprised 83.3% male GPs, and under Limitations/Discussion, that we were unable to make gender comparisons, as nuances based gender will require more extensive research to discern (lines 542-544).* |
| Previous query  *5) Results: Please include a discussion on the skewed gender distribution (25 males, 5 females) and its potential impact on findings.* | Our previous response  *The gender ratio of interviewed GPs has been addressed under Query G from Reviewer #1. Regarding the impact on the findings, we acknowledge the sample size being a limitation (Lines 542-544).* |
|  |  |

Once again, we are excited to publish these findings in a peer-reviewed journal, and PLOS Global Public Health would provide a suitable platform with its interest in TB as a global health topic. We also reiterate that our manuscript has not been submitted to any other journal.

We thank you for your kind consideration.

On behalf of the investigative team,

Yours sincerely,

**Jeremiah Chikovore, PhD.**

**Public Health Societies and Belonging (PHSB) division**

**Identity and Belonging Unit**
